# Ion Torrent Genexus as a Fast and Reliable Solution for HIV-1 Drug Resistance Testing: Comparison with the GeneStudio S5 Workflow

**DOI:** 10.3390/ijms27146307

**Published:** 2026-07-15

**Authors:** Flavia Smoquina, Federica Forbici, Giulia Berno, Martina Rueca, Alessandra Amendola, Giuseppe Sberna, Fabiano Brillo, Elisabetta Lazzari, Isabella Abbate, Gabriella Rozera, Silvia Sarti, Roberta Gagliardini, Valentina Mazzotta, Andrea Antinori, Fabrizio Maggi, Lavinia Fabeni

**Affiliations:** 1 Laboratory of Virology and Laboratories of Biosafety, National Institute for Infectious Diseases Lazzaro Spallanzani-IRCCS, 00149 Rome, Italy; flavia.smoquina@inmi.it (F.S.); federica.forbici@inmi.it (F.F.); martina.rueca@inmi.it (M.R.); alessandra.amendola@inmi.it (A.A.); giuseppe.sberna@inmi.it (G.S.); fabiano.brillo@inmi.it (F.B.); elisabetta.lazzari@inmi.it (E.L.); isabella.abbate@inmi.it (I.A.); gabriella.rozera@inmi.it (G.R.); silvia.sarti@inmi.it (S.S.); fabrizio.maggi@inmi.it (F.M.); lavinia.fabeni@inmi.it (L.F.); 2 Clinical and Research Department, National Institute for Infectious Diseases Lazzaro Spallanzani-IRCCS, 00149 Rome, Italy; roberta.gagliardini@inmi.it (R.G.); valentina.mazzotta@inmi.it (V.M.); andrea.antinori@inmi.it (A.A.)

**Keywords:** next-generation sequencing, HIV-drug resistance, Ion Torrent, Genexus, GeneStudio S5, minority variants, bioinformatic pipelines, turnaround time

## Abstract

Next-generation sequencing (NGS) has improved HIV-1 genotypic resistance testing (GRT) by enabling the detection of minority drug-resistance variants, although interpretation of low-frequency mutations remains challenging because of sequencing artifacts. This study compared the analytical performance and workflow efficiency of two Ion Torrent platforms, GeneStudio S5 (S5) and Genexus (GX), for routine HIV-1 GRT. A total of 134 plasma samples from people with HIV were prospectively collected, and 100 samples successfully sequenced on both platforms were included in the comparative analysis. Overall concordance for resistance-associated mutations was 88.0%, with agreement rates of 97.0% for protease, 90.0% for reverse transcriptase, and 100% for integrase. Both platforms generated clinically interpretable resistance profiles; however, 13 discordant mutations were identified. Application of a standardized confirmation algorithm, integrating Stanford HIVdb analysis with manual read-level inspection in Geneious software (version 2025.2.2), reclassified several discordant mutations as low-confidence or non-confirmed variants. Operationally, GX provided a fully automated workflow with approximately 24 h turnaround time and minimal hands-on processing, whereas S5 required approximately 72 h and substantially greater operator involvement. These findings support both platforms for routine HIV-1 GRT while emphasizing the importance of standardized bioinformatic review for reliable variant interpretation.

## 1. Introduction

In clinical care, research, and public health, the conventional HIV-1 genotypic resistance test (GRT) typically employs population-based Sanger sequencing (SS). However, this method struggles to reliably detect variants that occur at intra-host frequencies below approximately 20% [1]. Next-generation sequencing (NGS) can detect both high- and low-abundance drug resistance mutations (DRMs) and has demonstrated effectiveness in identifying HIV-1 DRMs [2,3]. SS is increasingly being replaced with NGS as a new standard for GRT during virological failure, at entry into care for individuals initiating antiretroviral treatment (ART), or during ART regimen switch [4]. Although HIV-1 genotypic resistance testing (GRT) is common in many high-income countries, it is not widely available everywhere. In low- and middle-income countries (LMICs), the implementation of GRT is still limited by infrastructure requirements, laboratory capacity, costs, and the need for specialized workers. Simplified and automated sequencing solutions may therefore represent an opportunity to expand access to molecular resistance surveillance and support individualized antiretroviral management in different healthcare settings [5,6].

Currently, several NGS platforms are available for HIV-1 drug resistance monitoring, including short-read sequencing technologies based on Illumina platforms, the Oxford Nanopore Technologies MinION, and the PacBio Sequel systems, which provide long-read sequencing capabilities [7,8,9]. Among these, Thermo Fisher Scientific Ion Torrent has been successfully introduced into our laboratory core infrastructure for routine diagnostics. This sequencing technology simplifies the NGS workflow with automatic library construction and templating by the Ion Chef System, followed by sequencing with the Ion GeneStudio S5 Systems (S5) (Thermo Fisher Scientific, Waltham, MA, USA), with a turnaround time of approximately three days [10,11].

However, the Ion Torrent platform, like all sequencing technologies, has specific technical limitations. The semiconductor detection method measures pH changes during nucleotide incorporation, making it particularly susceptible to errors in homopolymeric regions, portions of identical nucleotides where signal dephasing can lead to insertion or deletion errors. These technical artifacts can result in false-positive variant calls, especially in homopolymeric regions, which may be incorrectly classified as high-confidence low-frequency resistance mutations and could potentially lead to inappropriate clinical decisions [12,13,14,15].

Therefore, particular attention should be given to minority variants, especially those detected below 20% frequency, as these mutations require careful evaluation to distinguish low-frequency variants supported by manual read inspection from sequencing or bioinformatic artifacts. Manual inspection of read alignments using dedicated visualization tools and assessment of genomic context are recommended to support accurate interpretation of clinically relevant variants [16,17].

In late 2019, Thermo Fisher Scientific launched the new platform Ion Torrent “Genexus” (GX) Integrated Sequencer, which integrates library preparation, template preparation, and sequencing into a single-day, starting directly from RNA or DNA samples, reducing turnaround time, and minimizing interactions between the samples and the operator [18]. The GX platform employs enhanced chemistry and improved base-calling algorithms, including homopolymer-aware filtering, which may address the specific error profiles observed with earlier Ion Torrent systems [19].

The clinical laboratory setting imposes specific requirements on diagnostic platforms, including rapid turnaround times to support urgent therapeutic decisions, and minimal hands-on processing to reduce labour costs and the risk of errors. Despite increasing interest in fully integrated NGS systems, data on the performance of the GX platform for HIV-1 drug resistance testing in routine clinical settings are still limited, particularly in comparison with established workflows such as the S5. We report here a comparison of HIV-1 GRT in routine clinical practice using these two Ion Torrent NGS platforms at the National Institute for Infectious Diseases (INMI), “Lazzaro Spallanzani”, IRCCS Rome, Italy. The study evaluates analytical performance, workflow efficiency (turnaround time, hands-on time), and the clinical implications of platform-specific differences in minority variant detection and artifact generation.

## 2. Results

### 2.1. Sample Characteristics and Sequencing Performance

A total of one hundred and thirty-four plasma samples, from people with HIV (PWH), were collected, and one hundred plasma samples (74.6%) were successfully sequenced on both platforms and included in the comparative analysis. The remaining 34 samples were excluded due to failure on one or both platforms. The characteristics of the 34 samples not included in the analysis are described in Supplementary Table S1. To further investigate potential factors associated with unsuccessful sequencing, we compared the characteristics of samples successfully sequenced on both platforms with those that failed sequencing. These samples showed significantly lower viral loads than successfully sequenced samples (median 4.1 vs. 5.3 log_10_ copies/mL, *p* < 0.001), whereas no association was observed with HIV-1 subtype, CD4+ cell count, and treatment status. Notably, samples failing on both platforms (GX and S5) were characterized by particularly low viral loads (median 2.8 log_10_ copies/mL), suggesting that limited RNA input may contribute to unsuccessful sequencing results.

Overall, the population was predominantly male (73.0%), Italian (56.0%), and treatment-naïve (74.0%) subjects, with a median age of 44 years [interquartile range (IQR), 35 to 55 years]. Overall, at the time of sample collection, median viremia level and median CD4+ cell count were 5.24 log_10_ copies/mL (IQR, 4.80 to 5.85 log_10_ copies/mL) and 262 cells/mm^3^ (IQR, 106 to 377 cells/mm^3^), respectively.

Regarding the overall resistance profile of the study population, DRMs were detected across the main HIV-1 genomic regions analyzed, including protease (PR), reverse transcriptase (RT), and integrase (INT). According to the Stanford HIV Drug Resistance Database version 9.8 interpretation list (HIVdb) (https://cms.hivdb.org/prod/downloads/resistance-mutation-handout/resistance-mutation-handout.pdf, accessed on 20 June 2026), the overall prevalence of samples with at least 1 DRM was 42.0% in S5 and 43.0% in GX. In particular, PR DRMs were identified in 3.0% and 6.0% of samples analyzed by S5 and GX, respectively. RT DRMs were the most frequently detected, with nucleoside RT inhibitor (NRTI)-associated resistance mutations identified in 18.0% and 19.0% of samples by S5 and GX, respectively, while non-nucleoside RT inhibitor (NNRTI)-associated resistance mutations were detected in 26.0% of the analyzed samples by both platforms. Integrase inhibitor (INSTI)-associated resistance mutations were observed in 13.0% of cases.

Phylogenetic analysis confirmed correct clustering of S5 and GX sequences from the same individuals (bootstrap support > 90%) with no evidence of cross-contamination between samples. More than half (53.0%) of the individuals were infected with B subtype, followed by CRF02_AG and F1 (both 8.0%), A6 (4.0%), G, C and CRF93_cpx (all 3.0%). The remaining 18 individuals were infected with other pure subtypes or circulating recombinant forms (CRFs).

### 2.2. Concordance Analysis

Considering only DRMs, among the 100 samples, 88 showed complete aminoacidic level concordance across all three gene regions, while 12 samples showed discordant mutation profiles between the two platforms, including one sample in which two DRMs were detected (Table 1). Region-specific concordance varied: PR 97.0%, RT 90.0%, INT 100.0%.

### 2.3. Genexus and S5 Discordant Mutations

A subgroup of DRMs was detected exclusively by the GX platform at low frequencies (<20%), including F53L (*n* = 2) and N88D in the PR region and L74V, L100I, and K101E (*n* = 2) in RT (Table 1). According to HIVdb output, these variants were initially identified at frequencies ranging from 10% to 19%.

Following the predefined variant classification algorithm, described in Materials and Methods Section, all variants detected only by GX were re-evaluated by manual check of read alignments using Geneious Prime software. Overall, only a subgroup of these variants was confirmed as supported DRMs, while others were reclassified as low-frequency variants requiring caution or as non-confirmed. In particular, one F53L, one L74V and one K101E mutation were classified as supported, whereas the remaining mutations were not confirmed or considered low-confidence (Table 1).

Notably, these variants occurred predominantly in treatment-naïve individuals (71.4%, 5/7) and included mutations with potential or intermediate clinical relevance.

In contrast, a distinct group of RT DRMs, K65R, L74I, K101E, K103N (*n* = 2), and P225H was identified only by the S5 platform. After application of the same classification algorithm, most of these variants were confirmed at frequencies ≥10% and classified as supported DRMs, while one mutation was reclassified as low-frequency variants (Table 1). Of note, several discordant variants were located within or adjacent to homopolymeric regions (Supplementary Table S2), including both variants classified as supported and those not confirmed after manual inspection.

### 2.4. Workflow and Operational Comparison

**Figure 1:**
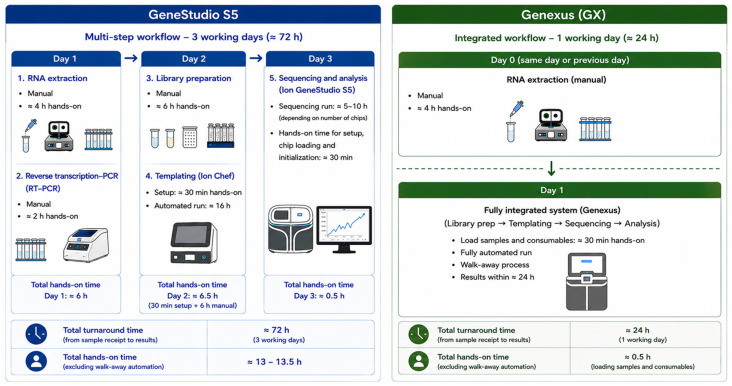
Workflow and performance comparison between S5 and GX platforms. Schematic representation of the two NGS workflows evaluated for HIV-1 GRT. The S5 workflow consists of multiple sequential steps, including RNA extraction, RT-PCR, library preparation, Ion Chef templating, sequencing, and data analysis, requiring approximately three working days from sample processing to final results. Differently, the GX platform integrates library preparation, templating, sequencing, and primary analysis into a single automated workflow, reducing operator intervention and providing results within approximately 24 h. The figure highlights the differences in workflow complexity, turnaround time, and operator involvement between the two platforms. This figure was generated with the assistance of an AI-based image-generation tool according to the authors’ specifications and was subsequently verified and refined by the authors. No third-party copyrighted images or schematic elements were incorporated.

The two platforms also showed clear differences in working performance (Figure 1). In particular, the S5 required approximately 72 h, corresponding to three working days, with manual RNA extraction (approximately 4 h of hands-on time) and RT-PCR (approximately 2 h of hands-on time) on the first day, library preparation (approximately 6 h of hands-on time) and templating on Ion Chef (30 min setup + 16 h automated) on the second day, sequencing and analysis on the third day. The sequencing step required approximately 5 to 10 h depending on the number of chips to be processed; however, only about 30 min of hands-on time was needed for instrument initialization and chip loading.

In contrast, the GX system completed the entire workflow within 24 h, with manual RNA extraction performed separately on the day before or same day, thereby providing a substantially faster end-to-end process. The GX platform required approximately thirty minutes of hands-on time for loading sample and consumable, as all subsequent processes were fully automated. The high level of automation provided by the GX platform reduces turnaround time, and also decreases operator-dependent variation, which is a known source of inconsistency in multi-step NGS workflows. This feature is particularly relevant in routine clinical laboratories, where reproducibility and standardization are critical. Compared with the multi-step open S5 workflow, the enclosed GX system limits sample and environmental exposure, thus reducing the risk of contamination.

## 3. Discussion

The transition from Sanger sequencing to NGS represents a fundamental change in HIV-1 drug resistance monitoring, addressing the critical limitation of conventional methods in detecting minority variant populations [20,21,22]. The present study provides a comparative evaluation of two Ion Torrent NGS platforms, S5 and GX, applied to HIV-1 GRT in a clinical diagnostic setting. Overall, both platforms demonstrated the ability to generate clinically interpretable resistance profiles, confirming their compatibility for routine HIV-1 GRT [11]. In particular, concordance between the two systems was high (88.0%), confirming that both platforms provide reliable HIV-1 genotypic resistance profiles across the protease, reverse transcriptase, and integrase regions. This observation is in line with previous studies showing that NGS-based HIV-1 GRT produces highly concordant results across different sequencing technologies when clinically relevant detection thresholds are applied [23]. Although most of the samples were successfully sequenced, 34 samples were excluded from the analysis because they failed on one or both sequencing platforms. Analysis of the excluded samples suggests that low viral load remains an important factor influencing sequencing success. In particular, concurrent failure of both platforms was predominantly observed in samples with very low viral loads (median 2.8 log_10_ copies/mL), supporting previous observations that limited RNA input may compromise amplification efficiency [24,25]. However, GX failures were observed across a broader range of viral loads, indicating that other factors than viral load alone may also influence assay performance. Therefore, although both platforms showed good overall analytical performance, sequencing success remains partially dependent on sample quality and viral RNA availability [24,25]. Further studies to investigate sequencing failures and operational performance will be necessary to better characterize the limitations of the GX workflow in routine clinical practice.

An important finding of the present study is that differences in resistance mutation detection were influenced not only by the sequencing platform itself but also by subsequent analysis. Following implementation of a predefined variant classification algorithm integrating HIVdb analysis, manual inspection of read alignments using Geneious software, and predefined frequency thresholds, several variants initially detected by only one of the two sequencing platforms were reclassified as low-confidence signals or were not confirmed. These findings support the concept that variant detection alone is insufficient to establish biological relevance, particularly for minority variants, and highlight the importance of combining automated bioinformatic analysis with expert manual review. Similar observations have been reported by Perrier and colleagues, who demonstrated that different bioinformatic pipelines may substantially influence the detection of low-frequency HIV resistance mutations even when identical sequencing data are analysed [26]. Another important observation was that the majority of discordant mutations were located within or adjacent to homopolymeric regions, which are known to represent a technical challenge for semiconductor sequencing technologies. Ion Torrent sequencing detects nucleotide incorporation through pH changes measured by ion-sensitive field-effect transistors [12,13], and in homopolymeric regions, particularly poly-A and poly-T stretches, incorporation of multiple identical nucleotides during a single flow cycle may generate signal intensity variations that complicate accurate base calling [27]. However, after manual inspection, we observed that both supported and non-confirmed variants could occur in homopolymeric contexts. Therefore, although these regions should be carefully monitored during HIV-1 drug resistance analysis, genomic context alone was not sufficient to explain the observed discordances or to distinguish true DRMs from low-confidence calls. The present study also highlights the importance of distinguishing between analytical validation and clinical interpretation. HIVdb represents the current reference standard for HIV drug resistance interpretation; however, it is not intended to validate the analytical reliability of variants generated by NGS pipelines. The developers of HIVdb-NGS explicitly acknowledge that minority variants are at increased risk of representing sequencing artefacts and that the current interpretation algorithm does not account for the threshold at which mutations are detected (https://hivdb.stanford.edu/hivdb/by-reads/, accessed on 20 June 2026). Our results are consistent with this observation, showing that several low-frequency discordant variants were reclassified after manual read-level inspection (Table 1). These findings support the integration of automated resistance interpretation with expert review of sequencing evidence to improve confidence in clinically relevant variant reporting. Furthermore, in laboratories with extensive experience in HIV GRT, interpretation should not rely only on the sequencing result. The integration of historical genotypic resistance profiles, previous virological data, and the patient’s antiretroviral treatment history represents an additional and essential step for assessing the biological acceptability and clinical relevance of detected mutations, particularly when low-frequency variants are identified [28]. This issue is particularly relevant in naïve individuals, in whom these DRMs, when confirmed, are indicative of transmitted drug resistance (TDR). Several studies have demonstrated that pre-treatment minority drug-resistant variants, particularly those harboring NNRTI resistance mutations, are associated with an increased risk of virological failure during first-line ART [15,20,29,30].

From an operational point of view, GX demonstrated clear advantages over S5. The fully integrated workflow substantially reduced operator intervention, required only approximately 30 min of hands-on time and generated complete sequencing results within approximately 24 h compared with approximately 72 h for the conventional S5 workflow (Figure 1) [31,32]. Besides reducing turnaround time, the closed automated workflow minimizes manual handling and may reduce operator-dependent variability and the risk of cross-contamination during multistep library preparation. These characteristics may be particularly advantageous in clinical situations requiring rapid therapeutic decision-making or when laboratory personnel and molecular expertise are limited. These operational benefits should nevertheless be interpreted alongside economic considerations. In our laboratory, the estimated cost per sample was approximately €210 for GX compared with approximately €150 for S5, depending on sequencing chip configuration (Supplementary Table S3). The higher cost of GX mainly reflects the fully integrated cartridge-based chemistry that combines reverse transcription, library preparation, templating and sequencing into a single consumable. On the contrary, the S5 workflow distributes costs across multiple steps, allowing greater flexibility in sample batching and chip utilization. Consequently, although GX provides clear workflow advantages, S5 remains a cost-effective solution for routine high-throughput laboratories processing larger sample numbers. Selection of the sequencing platform should also consider not only analytical performance but also laboratory organization, testing volume and clinical urgency. Additionally, as highly automated NGS technologies become increasingly adopted in clinical diagnostics, future reductions in reagent and consumable costs may improve their economic sustainability and further facilitate their implementation in routine laboratory settings [33].

Several limitations should be recognized. First, the study was performed exclusively using two Ion Torrent sequencing platforms without independent validation using an orthogonal sequencing technology, such as Illumina. Second, this was a single-centre study conducted within a routine diagnostic laboratory, and analytical performance may vary in other laboratory settings using different workflows or bioinformatic pipelines. Third, although minority variants were identified and classified according to standardized criteria, the present study did not evaluate their association with long-term clinical outcome or virological failure, which remains an area of ongoing investigation.

In conclusion, both S5 and GX represent reliable platforms for routine HIV-1 GRT. While GX offers substantial advantages in automation, reduced hands-on time and faster turnaround, S5 remains an accurate and more economically attractive solution for routine high-throughput testing. Moreover, our findings indicate that careful interpretation of low-frequency variants is essential regardless of the sequencing platform employed. The combination of standardized bioinformatic analysis with manual read-level confirmation provides a practical and transferable strategy to improve the reliability of HIV-1 GRT in routine clinical laboratories.

## 4. Materials and Methods

### 4.1. Samples and Viral Load

One hundred and thirty-four consecutive plasma samples from PWH received for routine clinical HIV-1 GRT were prospectively collected during 2024–2025 at INMI “Lazzaro Spallanzani”-IRCCS. Sample information, including date of sampling, viral load, nucleotide sequences, and detected mutations, together with demographic, clinical, and therapeutic data, was recorded in an anonymous database following institutional ethical guidelines. No additional selection criteria, other than plasma viral load ≥500 copies/mL, were applied, to ensure adequate RNA input for reliable NGS analysis. This threshold was selected based on previous validation studies demonstrating that samples with viral loads below this level may produce insufficient sequencing depth for accurate minority variant detection at the 10% threshold [11]. Plasma viral load was determined using the Aptima HIV-1 Quant Dx Assay, which runs on the fully automated Panther system (Aptima, Hologic, Inc., San Diego, CA, USA). The assay reports quantitative HIV-RNA results in the range of 30 to 10,000,000 copies/mL [34,35].

### 4.2. RNA Extraction

Viral RNA was manually extracted from 1 mL of plasma sample to increase the amount of extracted RNA, using the QiAmp Viral RNA mini kit ((QIAGEN, Hilden, Germany, 52904)). Plasma samples were first ultracentrifuged at 23,000× *g* for 120 min at 4 °C to pellet viral particles. The supernatant was discarded, and the viral pellet was resuspended in 560 μL of lysis buffer, and the lysate was subsequently processed for viral RNA extraction according to the manufacturer’s instructions [24]. This concentration approach maximizes RNA yield from clinical samples, which is critical for reliable detection of low-frequency variants that may represent a small fraction of the total viral population.

### 4.3. Next-Generation Sequencing: Ion GeneStudio S5 Prime

NGS sequencing, for routine clinical purposes, was first performed using the Ion S5 prime System by AmpliSeq primer pool (Thermo Fisher Scientific), which generated 17 overlapping amplicons spanning from PR to INT regions (pol region) [11]. Specifically, 7 µL of RNA, previously diluted according to viral load, was reverse-transcribed using NGS RT Kit (Thermo Fisher Scientific) in a 10 µL reaction. Two amplicon pools were then generated with 3 µL of cDNA using the Ion AmpliSeq Library Kit (Thermo Fisher Scientific), in a final volume of 10 µL per pool. These amplicon libraries were combined and digested with 2 µL FuPa reagent. Unique IonCode Barcode Adapters (Thermo Fisher Scientific) were ligated to each sample library. The libraries were purified using 40 µL Ampure XP magnetic beads (Beckman, Brea, CA, USA),washed twice with 70% ethanol, and eluted in Low TE buffer. Library quantification was performed using QUBIT (Thermo Fisher Scientific), normalized to 50 pM, and pooled in equimolar amounts. Template preparation of the 25 µL pooled library was automated using the Ion Chef Instrument (Thermo Fisher Scientific) with the Ion 510/520/530 Chef Kits and Ion 520/530 chips. Sequencing was conducted on the Ion S5, where twelve samples were processed per sequencing run, with each sample yielding approximately 500,000–1,000,000 reads. The complete workflow required three working days from sample receipt to sequence data generation.

### 4.4. Next-Generation Sequencing: Ion Torrent Genexus System

The same RNA extracts were subsequently sequenced using the GX platform. In particular, RNA samples were again diluted according to viral load. Briefly, 25 μL of each diluted sample was added to MicroAmp Optical 96-Well Clear Reaction Plates with Barcode (Thermo Fisher Scientific). Library preparation was performed using GX Barcodes 1–32 AS and Ion Torrent GX Library Strips 1 and 2-AS (Thermo Fisher Scientific), with one pair of library strips used for every four samples. Additionally, 75 μL of the same AmpliSeq two-primer pools, used for S5, were added into the GX Primer Pool Tube (Thermo Fisher Scientific). For templating, Ion Torrent GX Templating Strips 3-GX5 and 4 (Thermo Fisher Scientific) were used, with one pair of strips per chip lane. Sequencing was performed using the Ion Torrent GX Sequencing Kit, which includes the GX Cartridge, Bottles 1–3, the GX5 Chip, and the GX Coupler (Thermo Fisher Scientific). The GX system automatically calculated the required reagents and consumables, prompting the user to load them in designated positions within the deck chamber and reagent bay. Twelve samples were processed per run, generating approximately 1,300,000 reads per sample per lane on the four-lane GX5 chip. The entire process was completed within one day.

### 4.5. Variant Analysis and Classification Algorithm

FASTQ files generated from both sequencing platforms were analyzed using the Stanford HIV Drug Resistance Database (HIVdb, version 9.8), which was the main pipeline for variant detection and resistance interpretation. Notably, HIVdb is widely used in routine clinical laboratories for HIV-1 genotypic resistance testing, typically applying its default analysis parameters to ensure standardized and comparable results across different settings (https://hivdb.stanford.edu/hivdb/ngs2codfreq/, accessed on 20 June 2026). Preprocessing was therefore performed using the default HIVdb parameters, including adapter trimming, quality filtering, filtering of reads with excessive ambiguous bases, and minimum read length thresholds.

Reads were mapped to the HIV-1 HXB2 reference genome using a reference-based alignment approach relying on the Minimap2 algorithm. Codon frequency (codfreq) files generated within HIVdb were used for downstream variant detection and resistance interpretation. Variant calling was performed using standardized thresholds, including a minimum read depth of ≥100 reads and a mutation detection threshold of ≥10%, in line with clinical practice for HIV genotypic resistance testing. HIVdb output provided variant frequencies and corresponding resistance interpretation based on resistance mutation lists.

To further evaluate discordant variants, a secondary analysis was performed using Geneious (version 2025.2.2). In this step, Geneious was primarily used as a read-level visualization and confirmation tool rather than as an independent variant-calling pipeline, allowing manual inspection of read alignments and verification of variant frequency estimates. To ensure comparability between analytical approaches, we tried to reproduce, as closely as possible, the same alignment conditions implemented by the HIVdb pipeline within the Geneious software. In particular, read mapping in Geneious was performed against the same HXB2 reference genome using a Minimap2-based strategy with standard parameters for short-read data, including the use of secondary alignments and default alignment scoring. This approach was adopted to minimize methodological variability and ensure that any observed differences in variant detection were not attributable to discrepancies in the alignment step.

**Figure 2:**
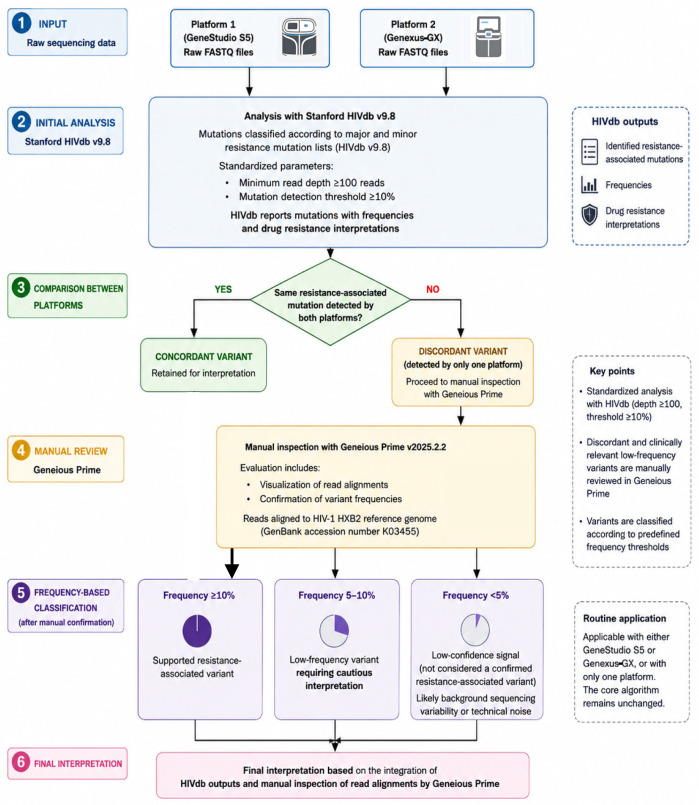
Analytical workflow and classification algorithm for HIV-1 drug resistance mutations. FastQ files were analyzed using Stanford HIVdb v9.8 with standardized thresholds (≥100 reads; ≥10% frequency). Concordant variants were retained, while discordant variants were manually reviewed in Geneious software. Variants were classified based on confirmed frequencies: ≥10% (supported), 5–10% (low-frequency, caution), and <5% (not confirmed). Final interpretation integrates automated HIVdb output with manual read-level validation. This figure was generated with the assistance of an AI-based image-generation tool according to the authors’ specifications and was subsequently verified and refined by the authors. No third-party copyrighted images or schematic elements were incorporated.

Moreover, a predefined variant classification algorithm (Figure 2) was applied to distinguish supported variants from low-confidence signals potentially related to technical variability. When the same resistance-associated mutation was identified by both sequencing platforms, the variant was considered concordant and retained for interpretation. Conversely, variants detected exclusively by one platform were considered discordant and underwent manual inspection using Geneious. After manual review, variants were classified according to predefined frequency thresholds, and variants detected at a frequency ≥10% were considered supported DRMs. Variants detected between 5% and 10% were classified as low-frequency variants requiring cautious interpretation, whereas variants detected below 5% were considered low-confidence signals and were not classified as confirmed, as they were considered potentially related to background sequencing variability or technical noise. This classification algorithm was applied independently of the sequencing platform used and can also be adopted in routine diagnostic settings where only one sequencing platform is available.

### 4.6. Phylogenetic Analysis

A phylogenetic analysis of HIV-1 pol sequences was performed for the following purposes: (i) to determine viral subtypes, (ii) to check for any cross-contamination, and (iii) to evaluate the proper clustering of the S5 and GX sequences from the same subjects. Briefly, the pol sequences were aligned with HIV-1 reference sequences of all subtypes from the Los Alamos National Laboratory HIV Database (https://www.hiv.lanl.gov/content/sequence/NEWALIGN/align.html, accessed on 20 June 2026).) using MAFFT version 7 (https://mafft.cbrc.jp/alignment/server/index.html, accessed on 13 April 2026). The alignment was edited using the BioEdit program, version 7.0.5.3. MEGA12 was used to perform phylogenetic analysis [36]. A phylogenetic tree was constructed with a neighbor-joining (NJ) method. The reliability of the branching orders was assessed by bootstrap analysis of 1000 replicates, as previously described [37].

### 4.7. Statistical Analysis

Differences between successfully sequenced and failed samples were assessed using the Mann–Whitney U test for continuous variables and Fisher’s exact test for categorical variables (https://astatsa.com/). A *p*-value < 0.05 was considered significant.

## Figures and Tables

**Table 1:** Comparison of discordant HIV-1 DRMs detected by the GX and S5 platforms.

Sample	Gene	Mutation	HIVdb Interpretation	Detection Platform	HIVdb Frequency (%)	Geneious Frequency (%)	Final Classification
1	PR	F53L	Potential reduced susceptibility to ATV	GX	10.0	14.1	Supported
2	PR	F53L	Potential reduced susceptibility to ATV	GX	13.0	3.9	Not confirmed
3	PR	N88D	Potential reduced susceptibility to ATV	GX	19.0	1.7	Not confirmed
4	RT	L74V	Intermediate resistance to ABC	GX	19.0	17.4	Supported
5	RT	L100I	Resistance to NNRTI	GX	13.0	9.6	Low-frequency variant (caution)
6	RT	K101E	Low-level resistance to EFV + ETR; intermediate resistance to NVP + RPV	GX	11.0	10.4	Supported
7	RT	K101E	Low-level resistance to EFV + ETR; intermediate resistance to NVP + RPV	GX	17.0	3.3	Not confirmed
8 ^a^	RT	K65R	Low-level resistance to FTC + 3TC; intermediate resistance to ABC + TFN	S5	86.0	69.2	Supported
8 ^a^	RT	L74I	Low-level resistance to ABC	S5	45.0	86.2	Supported
9	RT	K101E	Low-level resistance to EFV + ETR; intermediate resistance to NVP + RPV	S5	20.0	51.1	Supported
10	RT	K101E	Low-level resistance to EFV + ETR; intermediate resistance to NVP + RPV	S5	21.0	8.9	Low-frequency variant (caution)
11	RT	K103N	High-level resistance to EFV + NVP	S5	22.0	23.0	Supported
12	RT	P225H	Potential low-level resistance to DOR; intermediate resistance to EFV + NVP	S5	23.0	46.5	Supported

DRMs are reported according to genomic region (protease [PR] and reverse transcriptase [RT]), mutation, HIVdb interpretation, detection platform, and frequencies obtained by HIVdb and Geneious software. By a predefined confirmation algorithm, variants with Geneious frequency ≥10% were considered supported; variants between 5 and 10% were considered low-frequency variants requiring caution; variants < 5% were not confirmed. ATV: atazanavir; ABC: abacavir; FTC: emtricitabine; 3TC: lamivudine; EFV: efavirenz; NVP: nevirapine; ETR: etravirine; RPV: rilpivirine; DOR: doravirine. ^a^ Sample 8 showed two different RT DRMs (K65R and L74I). The color coding is used to indicate the level of evidence associated with each result: red denotes a not confirmed result, green indicates a supported result, and yellow identifies a low-frequency variant that should be interpreted with caution.

## Data Availability

The HIV sequence data generated in this study have been deposited in the NCBI BioProject database under accession number PRJNA1484951.

## Supplementary Materials

The following supporting information can be downloaded at: https://www.mdpi.com/article/10.3390/ijms27146307/s1.
