# Supplementary material for: Ion Torrent Genexus as a Fast and Reliable Solution for HIV-1 Drug Resistance Testing: Comparison with the GeneStudio S5 Workflow
Source: Int J Mol Sci. 2026 Jul 15;27(14):6307. doi: 10.3390/ijms27146307 (PMC13410318; doi:10.3390/ijms27146307)
Supplement: Supplementary file 1 [file ijms-27-06307-s001.zip › ijms-4376630-supplementary.pdf]

**Supplementary Table S1.** Characteristics of samples excluded from the comparative analysis due to sequencing failure on one or both platforms. The table reports treatment status (naïve or treatment-experienced), HIV-1 subtype, plasma viral load ($log_{10}$ copies/mL), CD4+ cell count (cells/mm$^3$), and the sequencing platform on which the sample failed. Samples indicated as GX+S5 failed sequencing on both platforms, whereas GX or S5 indicate failure exclusively on the corresponding platform. NA: not available.

| Sample | Naïve | Subtype | Viral Load ($log_{10}$cp/mL) | CD4 (cell/mm$^3$) | Failure platform |
|---|---|---|---|---|---|
| 1 | Yes | B | 3.4 | 926 | GX+S5 |
| 2 | No | B | 6.1 | 140 | GX |
| 3 | No | B | 4.6 | 45 | GX |
| 4 | No | A6 | 4.8 | 358 | GX |
| 5 | Yes | CRF12_BF | 6.6 | 13 | GX |
| 6 | Yes | CRF02_AG | 4.6 | 542 | GX |
| 7 | No | B | 3.5 | 241 | GX+S5 |
| 8 | Yes | G | NA | NA | GX |
| 9 | No | F1 | 3.4 | 403 | S5 |
| 10 | No | B | NA | NA | GX |
| 11 | Yes | B | 3.3 | 686 | GX |
| 12 | Yes | CRF06_cpx | 6.3 | NA | GX |
| 13 | Yes | CRF60_BC | 4.6 | 457 | GX |
| 14 | No | B | 2.9 | 329 | GX |
| 15 | No | B | 2.8 | 607 | GX+S5 |
| 16 | Yes | B | 4.9 | 142 | GX |
| 17 | No | B | NA | NA | GX |
| 18 | No | B | 2.9 | 565 | GX+S5 |
| 19 | Yes | B | 6.7 | 440 | GX |
| 20 | Yes | B | 5.0 | 843 | GX |
| 21 | Yes | CRF02_AG | 5.7 | 334 | GX |
| 22 | No | B | 4.4 | 264 | GX |
| 23 | No | CRF72_BF1 | 2.8 | 138 | GX+S5 |
| 24 | No | B | 2.7 | 775 | GX+S5 |
| 25 | Yes | CRF02_AG | NA | NA | GX |
| 26 | No | B | NA | NA | GX |
| 27 | No | B | 2.8 | 701 | GX+S5 |
| 28 | No | CRF60_BC | 2.9 | 272 | GX+S5 |
| 29 | Yes | B | 2.7 | 140 | GX+S5 |
| 30 | No | F1 | 3.3 | 545 | GX |
| 31 | No | B | 2.8 | 764 | GX+S5 |
| 32 | No | B | 6.5 | 22 | GX |
| 33 | Yes | CRF02_AG | 5.7 | 334 | GX |
| 34 | No | CRF71_BF | 4.1 | 398 | GX |

**Supplementary Table S2. Characteristics of discordant HIV-1 drug resistance mutations detected by GeneStudio S5 and Genexus.**

For each discordant mutation, the table reports position coverage obtained from the HIVdb and Geneious analyses, plasma viral load ($log_{10}$ copies/mL), HIV-1 subtype, treatment status, and the presence or absence of a homopolymeric context. Homopolymer context indicates whether the mutation was located within or adjacent to a homopolymeric region. The corresponding nucleotide sequence surrounding each mutation is reported in the Genomic region column. Coverage values represent the number of reads supporting the analyzed codon position in the respective analytical pipelines.

*Homopolymeric regions were defined by manual inspection of the HXB2 reference sequence and were identified as genomic regions containing stretches of ≥4 identical consecutive nucleotides surrounding the reported mutation position. NA: not available. [a]Sample 8 harbored two discordant resistance-associated mutations (K65R and L74I) and is therefore reported twice.

| Sample | Mutation | Position Coverage HVIdb (number of reads) | Position Coverage Geneious (number of reads) | Viral Load ($log_{10}$ cp/mL) | Subtypes | Naïve | Homopolymer context* | Genomic region |
|---|---|---|---|---|---|---|---|---|
| 1 | F53L | 8,474 | 8,167 | 6.0 | B | Yes | Yes | AATTGGAGGT**TTT**ATCAAAGTA |
| 2 | F53L | 5,493 | 2,458 | 7.3 | CRF40_BF | Yes | Yes | AATTGGAGGT**TTT**ATCAAAGTA |
| 3 | N88D | 6,564 | 5,686 | 5.4 | CRF47_BF | Yes | No | AATTGGAAGA**AAT**CTGTTGAC |
| 4 | L74V | 5,759 | 6,323 | 6.5 | B | Yes | Yes | ATGGAGAAAA**TTA**GTAGATTTCA |
| 5 | L100I | 34,131 | 31,398 | 5.5 | CRF23_BG | Yes | Yes | TCCCGCAGGG**TTA**AAAAAGAAAA |
| 6 | K101E | 107,065 | 11,118 | 4.1 | B | No | Yes | CGCAGGGTTA**AAA**AAGAAAAAAT |
| 7 | K101E | 601 | 271 | 5.3 | CRF93_cpx | No | Yes | CGCAGGGTTA**AAA**AAGAAAAAAT |
| 8[a] | K65R | 968 | 5,934 | 4.4 | C | No | Yes | TGCCATAAAG**AAA**AAAGACAGTA |
| 8[a] | L74I | 1,295 | 3,204 | 4.4 | C | No | Yes | ATGGAGAAAA**TTA**GTAGATTTCA |
| 9 | K101E | 11,303 | 4,639 | 4.6 | CRF93_cpx | Yes | Yes | CGCAGGGTTA**AAA**AAGAAAAAAT |
| 10 | K101E | 57,919 | 56,891 | 5.5 | B | Yes | Yes | CGCAGGGTTA**AAA**AAGAAAAAAT |
| 11 | K103N | 4,684 | 4,554 | 2.8 | B | No | Yes | GTTAAAAAAG**AAA**AAATCAGTAA |
| 12 | P225H | 4,505 | 202 | NA | CRF02_AG | No | No | TCAGAAAGAA**CCT**CCATTCCTTT |

**Supplementary Table S3.** Comparison of per-sample costs (expressed in euros) for the two sequencing platforms. Costs include all consumables required from extraction to sequencing result generation. For GeneStudio S5, library preparation and sequencing steps are performed as separate procedures, whereas Genexus integrates these steps into an automated workflow. Costs are calculated based on the laboratory workflow implemented in this study and may vary depending on batch size, reagent availability, and local procurement conditions. *Based on 520 or 530 chip.

| | Gene Studio S5 Prime | Genexus Integrated sequencer |
|---|---|---|
| **Extraction** | 5.4 | 5.4 |
| **Retrotranscriptase** | 4.7 | 172.85 |
| **Library Preparation** | 83.77 | |
| **Sequencing (using 520 chip)** | 53.12 | |
| **Sequencing (using 530 chip)** | 58.1 | |
| **Accessory reagents (plasticity and quantization)** | 0.75 | 33.25 |
| **Total cost per sample** | 147.74 or 152.72* | 211.5 |
